# Surface Morphology and Tooth Adhesion of a Novel Nanostructured Dental Restorative Composite

**DOI:** 10.3390/ma9030203

**Published:** 2016-03-16

**Authors:** Marco Salerno, Patrizia Loria, Giunio Matarazzo, Francesco Tomè, Alberto Diaspro, Roberto Eggenhöffner

**Affiliations:** 1 Istituto Italiano di Tecnologia (IIT), via Morego 30, I-16163 Genova, Italy; marco.salerno@iit.it (M.S.); alberto.diaspro@iit.it (A.D.); 2 Dipartimento di Scienze Chirurgiche e Diagnostiche Integrate—Scuola di Medicina—Università di Genova, corso Europa 30, I-16132 Genova, Italy; patrizia.loria@unige.it; 3 Private practice, MD, DDS, Dental Office in Genova, via Rimassa 51, I-16129 Genova, Italy; matarazzo.giunio@libero.it; 4 Private practice, MD, DDS, Dental Office in Mestre, corso del Popolo 111, I-30125 Mestre (VE), Italy; tf_studio@libero.it; 5 INBB Istituto Nazionale Biostrutture e Biosistemi, viale delle Medaglie d’Oro 305, I-00136 Roma, Italy

**Keywords:** dental resin composite, nanoporous filler, anodic porous alumina, atomic force microscope, adhesion strength

## Abstract

Recently, a novel dental restorative composite based on nanostructured micro-fillers of anodic porous alumina has been proposed. While its bulk properties are promising thanks to decreased aging and drug delivery capabilities, its surface properties are still unknown. Here we investigated the surface morphology and the adhesion to tooth dentin of this composite as prepared. For comparison, we used two commercial composites: Tetric EVO Flow (Ivoclar) and Enamel HRi Plus (Micerium). The surface morphology was characterized by atomic force microscopy and the adhesion strength by tensile tests. The experimental composite is rougher than the commercial composites, with root mean square roughness of ~549 nm against 170–511 nm, and presents an adhesion strength of ~15 MPa against 19–21 MPa. These results show at the same time some proximity to the commercial composites, but also the need for optimization of the experimental material formulation.

## 1. Introduction

Resin composites are the most adopted materials in the treatment and prevention of dental pathologies [1]. Nevertheless, certain properties of dental composites still need to be optimized, *i.e.*, mechanical strength, biocompatibility and surface characteristics [2,3]. The surface properties are amongst the most studied object of current scientific research in the field of dental composites since they are directly associated with the immediate success and lifetime of restoration [4]. In particular, in the oral environment, the surface roughness of the restorative composites favors the adsorption of salivary proteins, the adhesion of bacteria and the formation of the bacterial plaque, which causes infiltration and secondary caries [5,6]. A large surface having high roughness and waviness can hardly be cleaned in an optimal manner during the dental hygiene operation of tooth-brushing [7]. Additionally, the roughness of dental composites is associated with degradation of the esthetical qualities of any restoration. As pointed out in recent works, nanoscale characterization of these surfaces is of great importance [8,9,10]. Furthermore, the surface roughness critically affects the integration and bonding of the restorative composite to the dental tissue cavity, which results in improved adhesion strength.

The use of anodic porous alumina (APA) [10] as a filler in dental resins was conceived to enhance the long-term stability in mechanical performances and limit the elution of chemical degradation products in the mouth, occurring in standard composites after hydrolysis of the bonding agent. In a previous work [11], simulated aging showed for the experimental APA-based composite (EAC) a decrease in storage modulus of maximum ~20% as compared to the typical decrease of ~50% for two commercial composites selected for reference. Additionally, a possible drug delivery function was demonstrated. Given these promising results, as the next step towards a full testing of the requirements for possible clinical use of the EAC, the proper adhesion to dental tissues was to be checked. The present study compares the surface morphology and the adhesion to dentin of the EAC to two commercial composites, Tetric EVO Flow (TEF) (Ivoclar) and Enamel HRi Plus (EHP) (Micerium). The EAC investigated here is identical to the previously investigated material. The null-hypothesis is that there is no difference between the EAC and the tested commercial composites in surface roughness and bond strength to dentin.

## 2. Results and Discussion

**Figure 1:**
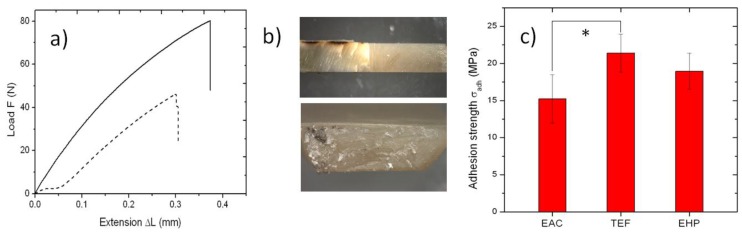
(**a**) Example of raw data obtained during tensile tests. In some cases (**dotted curve**) the specimen was partly slipping between the clamps, especially at the beginning of pulling; while this did not affect the maximum force measured on breaking, we discarded experiments with significant slipping; (**b**) typical optical images (not at same scale) of side view of the tooth composite specimens used (top), and broken interface in cross section (bottom); (**c**) bar plot (means ± one standard deviation) of the adhesion strength for all the three composites considered. The asterisk represents statistically significant differences, (*: *p* < 0.05).

In this work, we measured for the first time the strength of adhesion of the EAC to dentin, as shown in Figure 1. When pulling specimens such as in Figure 1b top, load-extension curves were obtained as the raw data similar to those shown in Figure 1a for the EAC, which broke at the dentin-composite interface, leaving a fracture surface as in the bottom of Figure 1b. In the plots of Figure 1a, for the horizontal axis, it was not possible to accurately evaluate the initial thickness and thus the occurring relative extension (strain) of the EAC-tooth interface layer. Additionally, as a side effect, the slipping of some specimens inside the clamps occasionally occurred, mainly at the beginning of the tests (see the dashed curve in Figure 1a). This effect affected also the possible information on the elastic modulus of the interface layer, which is associated with the slope of the curves. However, the information of interest to be extracted from these plots is the maximum stress at break σ_t_, which was obtained by dividing the maximum load reached by the area of the cross-section resulting from the fracture (Figure 1b bottom).

A summary of the results of tensile tests for all composites, after removing the curves affected by significant slipping, is reported in Figure 1c. The values obtained, expressed as mean ± one standard deviation, are 15.2 ± 3.3 MPa for the EAC, 21.4 ± 2.6 MPa for TEF and 19.0 ± 2.4 MPa for EHP. *n* = 8, 6, and 6, for EAC, TEF, and EHP, respectively. Notably, all these values are lower than the average ones occurring in the literature for other optimal working specimens of well-tested commercial composites, which typically fall in the range of 20–60 MPa (see e.g., Ref. [12]). Part of the observed deviation towards lower values may be due to degradation of the collagenous fibrils resulting from etching dead teeth not optimally preserved, or from problems in alignment of the specimens along a single straight axis (uniaxial-testing) of the pulling clamps. Additionally, a size effect may also have affected our estimations, since we used typical cross‑section area of the pulled interfaces higher than those recommended in the literature [12], and it is known that smaller bond areas may exhibit higher bond strength due to less likely occurrence of critical defects and better distribution of stress, in agreement with Griffith theory [1].

In particular, what is most important here is the comparison between the EAC and the commercial composites. After ANOVA, it appears that EAC and EHP are not significantly different, even at the lowest level of significance (*p* > 0.05), whereas EAC presents significantly lower adhesion strength than TEF (*p* < 0.05). However, the difference was not very significant (*p* > 0.01). Therefore, at least in the case of EHP, the adhesion performance is similar to EAC. On the other hand, EAC shows the lowest mean and also the highest standard error (ratio of standard deviation to mean), namely 1.45 *versus* 1.15 of TEF and 1.10 of EHP. This higher spread may represent a lower reproducibility in values. Therefore, there is room for improvement in the adhesion properties of the EAC, by increasing its homogeneity and possibly by fine tuning in the formulation.

The different adhesion of the composites to the dental tissue can be probably characterized further by investigating their intrinsic surface roughness, as resulting from standard polishing procedure. In fact, the surface characteristics of a loaded resin may vary depending on the nature and methods of inserting the fillers. Whereas TEF is a flowable, hybrid composite, containing a mixture of micro-coordinated inorganic fillers and glass fillers with broad multi-disperse size distribution around an average size of ~1 μm [1,13,14,15], EHP is a universal composite for bulk restorations, based on nano-particles and known for its good aesthetics and high abrasion resistance [16]. The surface roughness, in particular, is one major parameter in dental restorative materials: an excess roughness may not allow conformal wetting of dental surfaces and will thus weaken the adhesion of materials to their natural support. Furthermore, roughness favors the growth of micro-organisms in the oral environment [5,6] and detrimentally affects through scattering aesthetical parameters to be also evaluated in view of the actual industrial applicability of materials.

**Figure 2:**
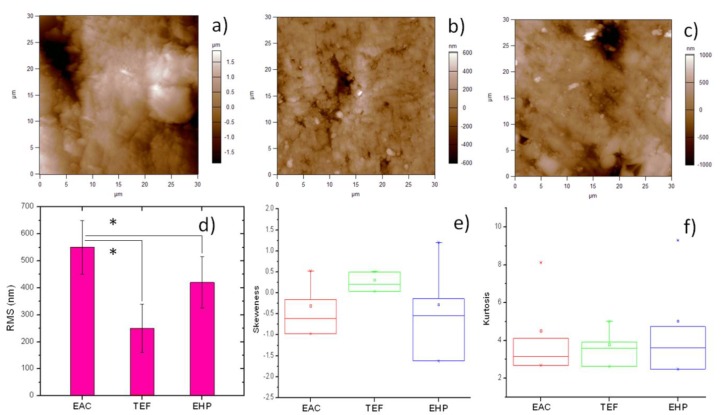
Examples of typical 3D surface morphology resulting from 30 µm AFM scan size of the different composites after standard polishing: (**a**) EAC; (**b**) TEF; and (**c**) EHP; (**d**–**f**) statistical plots of the quantities of interest extracted from AFM figures such as in Figure 2, namely (**d**) RMS roughness S_q_ (bar plot); (**e**) skewness S_sk_; and (**f**) kurtosis S_ku_, respectively (box plots). For S_q_ in (**d**), the asterisk represents statistically significant differences, (*: *p* < 0.05).

Atomic force microscopy (AFM) is considered today the golden standard for evaluation of 3D morphology of material surfaces on the micro-scale and down to nano-sized resolution [8,17]. Representative AFM images showing the typical surface morphology of the investigated composites are shown in the upper row of Figure 2. Given the scan size of 30 μm, the single pixel size was ~60 nm. It appears that the EAC presents both valleys and protrusions of large size, comparable to the microscale fillers (such as the square platelet on the mid-right in Figure 2a). The nanopores in the filler are not visible, probably due to the resin matrix filling and flattening them, as shown in Ref. [11] based on stiffness contrast mapping. Similar large fillers are not visible in TEF (Figure 2b) and EHP (Figure 2c). The former in particular seems to exhibit the smaller and more rarely sparse particles. Overall, while in EHP (Figure 2c) some lay due to polishing appears, still the surface with the less regular surface pattern is that of EAC (Figure 2a).

The above considerations are supported by the quantitative analysis. The roughness amplitude parameters of RMS S_q_, skewness S_sk_ and kurtosis S_ku_ of the step-height distributions, as resulting from the whole sets of images (*n* = 12 for each material) are reported in the lower row of Figure 2. In particular, for S_q_, we obtained a value of 260 ± 90 nm for TEF, 418 ± 93 nm for EHP, and 549 ± 98 nm for EAC (see Figure 2d). The ANOVA showed that there is no significant difference in S_q_ between the two commercial composites, whereas EAC shows a significant difference (*p* < 0.05) with respect to both of them. Therefore, EAC scores lower than both commercial composites in the S_q_ surface roughness parameter. However, the relative difference is not huge, especially with respect to EHP, and in no case is it very significant (*p* > 0.01).

It is also observed that, between the two commercial composites, TEF exhibits higher adhesion and lower roughness. Thus, both effects are probably associated with the presence in TEF of fillers that are both smaller in average size (nano-filled material) and lower in quantity, as known for flowables in comparison to packable or universal composites, which usually limits their application to small restoration or lining the prepared cavities [18]. These data are in favor of a real correlation between the two above properties of roughness and adhesion.

While S_q_ is the most commonly used amplitude parameter describing the surface roughness also in the areas of biomedicine and dentistry [19,20,21], additional amplitude parameters may also help to describe the surface topography of our composites. In Figure 2e, the results for S_sk_ are presented. This quantity qualifies the symmetry of the surface since negative (positive) values indicate predominant valleys (peaks). In this respect, EAC is similar to EHP, with broad distribution shifted towards negative values (valleys). TEF, on the contrary, has a more narrow distribution of S_sk_ and slightly shifted towards positive values: in the composite filled with smaller particles and at lower levels, obviously there appear no valleys between the fillers. Finally, S_ku_ (Figure 2f) qualifies the type of surface roughness, being > (<) 3 for spiky (bumpy) surfaces. In this respect, the composites are all similar, rather balanced between the two above cases, and resemble almost perfectly random surfaces (S_sk_ = 3), especially for EAC.

In conclusion, under the limitations of the present study (tensile clamps not optimal and relatively low statistics), the null-hypothesis is that there is no difference between the EAC, and the tested commercial composites in surface roughness and bond strength to dentin has been rejected. This indirectly confirms the issues of irregular surface morphology of the EAC, which probably affects the intimate contact and bonding of the composite to the dental cavities. However, the differences with the commercial composites, both in roughness and adhesion, are significant (*p* < 0.05) but not very significant (*p* > 0.01). In conclusion, the present analysis of the EAC suggests that, while the EAC composite is probably not valuable as a universal restorative material in its current formulation, it can be either appropriately modified or used for different applications, still retaining its advantages of aging stability and potential nanopores loading. Possible modifications in the EAC formulation could be including also smaller APA fillers to fill the gaps between the ~5 µm large ones, without losing the advantage of the nanoporous APA structure *i.e.*, making them of the order of 0.5–1 µm, or adopting a hybrid heterogeneous filler formulation with nanosized (non-porous) fillers intermixed with the nanoporous APA micro-fillers, which would probably allow to increase the loading to the levels of commercial materials (70–80 wt %), thus reaching even higher elastic modulus up to ~10 GPa.

## 3. Materials and Methods

### 3.1. APA Fillers

High purity (>99%) Al foils with 250 μm thickness (Goodfellow, Cambridge, UK) were coated on one side with special protective paint [22] and anodized according to a previously described protocol [23] in a double-walled beaker cooled by circulating liquid at +10 °C. Even with single-step anodization, while the pores are not regularly arranged in a hexagonal array, the pore size is relatively monodisperse (coefficient of variation ≤ 13%) and uniform over a distance of several centimeters [24]. The oxide coating was set free by dissolving the paint in acetone and the aluminum substrate in CuCl_2_ [23]. Under the selected conditions, after opening the pores in phosphoric acid [25], a mean pore diameter of ~80 nm and interpore distance of ~150 nm were obtained. The APA membranes were then ball-milled as described elsewhere [11]. The APA powder obtained had particles of 5–10 μm diameter, as resulting from scanning electron microscope inspection.

### 3.2. Resin Composites

The two commercial restorative composites, namely Tetric EVO Flow (Ivoclar Vivadent, Liechtenstein) and Enamel HRi plus (Micerium, Italy), shortly called TEF and EHP in the following, came in 3 g syringes and were used according to the manufacturers’ specifications. Irradiation was carried out with a blue lamp LED.B (Carlo de Giorgi, Italy) of nominal power 1 mW, on layers of approximately 1 mm thickness, each undergoing 30 s exposure in order to obtain a complete photopolymerization.

The EAC was prepared from a 70:30 (wt) mixture of co-monomers 2,2′-bis[4-3-(methacryloxy- 2-hydroxy-propoxy)-phenyl]-propane (Bis-GMA) and triethylene glycol dimethacrylate (TEGDMA), the latter used to decrease the viscosity. To this paste, a photoinitiation system based on dimethyl amino ethyl methacrylate (DMAEMA) and Camphorquinone (CQ), prepared separately, was added, each as 0.5% of total co-monomer. The organic paste was loaded by sonication-assisted spatulation with 50 wt % APA powder without silane bonding agent and then irradiated under the same conditions as the commercial reference materials. In Table 1, the list of all the EAC ingredients is given, with respective sources and amounts.

### 3.3. Sample Preparation

For surface morphology analysis by AFM, the three composites were polymerized in the form of discs of ~1 mm thickness (6 specimens for each sample were fabricated). Then, the specimens were polished by rotating grommets with descending diamond particle size [20].

For tensile tests of adhesion to tooth tissue, a total of 12 first molar or premolar teeth, free of caries and previous restorations, were extracted for orthodontic reasons and stored in physiological solution. The experimentation on human tissue has been carried out in accordance with ethical principles. All subjects enrolled have responded to an Informed Consent. The apical crown of the teeth was cut off by a circular diamond saw rotating at 500 rpm under a jet of distilled water. The exposed cross section was treated with a standard etching and adhesion system (scotchband universal adhesive, (3M ESPE, St Paul, MN, USA). Then, a cylindrical crown of either composite was built up on top of the cut surface, in 1 ± 0.2 mm increments. Finally, the tooth-composite body was cut into a parallelepiped and then cut perpendicular to the tooth-composite interface, such as to make half tooth-half composite specimens of ≥ 10 mm length, 1–2 mm thickness, and ~3–6 mm width, (see Figure 1b top). A total of 14, 12, and 11 specimens were successfully prepared for EAC, TEF and EHP, respectively.

#### 3.3.1. Tensile Tests

The specimens obtained from the tooth-composite cutting for all the different composites have been tested under tensile mode in a universal testing machine 3365 (Instron, High Wycombe, UK) at cross-head speed of 1 mm/min. The raw data of elongation and force were collected continuously, and the maximum force reached at break was recorded (see e.g., Figure 1a). From the starting sets of specimens, 6, 6 and 5 specimens failed to break at the tooth-composite interface, for EAC, TEF, and EHP, respectively. (Of these, 4, 3, and 3 broke in the tooth side, and the rest in the composite side, for EAC, TEF, and EHP, respectively). As a consequence, *n* = 8, 6, and 6, for EAC, TEF, and EHP, respectively. The cross section of the specimens apparently broken at the tooth-composite interface was inspected in a stereomicroscope EZ series (Leica Microsystems, Wetzlar, Germany), to evaluate the area of the broken cross-section, by means of scale-calibrated images (see e.g., Figure 1b bottom). This area was used to calculate the maximum stress reached on breaking, which was assigned as the value of adhesive strength *σ_t_*. An example of raw data plots is shown.

#### 3.3.2. AFM Characterization

The AFM instrument MFP-3D (Asylum Research, Santa Barbara, CA, USA) was used, with NCHR (NanoWorld, Neuchâtel, Switzerland) probes in tapping mode in air. These silicon probes have a nominal resonance frequency and spring constant of ~320 kHz and ~42 N/m, respectively. The tip is a pyramid with ~13 µm length, ~20 nm apex diameter, and 2:1 tip aspect ratio. For each specimen, 2 areas of 30 × 30 µm^2^ were scanned, represented with 512^2^ pixels. The images obtained were processed with the AFM company software Version-12, based on IgorPro 6.22 (Wavemetrics, Lake Oswego, OR, USA). The surface roughness and morphology (data-points *n* = 2 images/specimen × 6 specimens/sample = 12) have been described based on the distribution of step-heights RMS, *i.e.*, S_q_; the third statistical moment, *i.e.*, skewness S_sk_; and the fourth statistical moment, *i.e.*, kurtosis S_ku_ [26].

#### 3.3.3. Statistical Analysis

On both tensile strength *σ_t_* and RMS roughness S_q_, analysis of variance (ANOVA) has been carried out by means of software Origin 8.1 (OriginLab, USA), to assess the statistical significance of the apparent difference of values for the three composites, with different levels of significance (0.05 and 0.01) and pair comparisons based on Tukey tests.

## Figures and Tables

**Table 1:** List of all the ingredients (in short names) of the experimental APA-based composite (EAC).

Ingredient	Manufacturer	Lot #	Amount (wt %)
Bis‑GMA	Sigma-Aldrich (Italy)	MKBD8328	34.653
TEGDMA	Sigma-Aldrich (Italy)	BCBC5367V	14.851
DMAEMA	Sigma-Aldrich (Italy)	1437599V	0.248
CQ	Sigma-Aldrich (Italy)	S12442	0.248
APA filler	home made	–	50.000

## Abbreviations

The following abbreviations are used in this manuscript:

APA	anodic porous alumina
EAC	experimental APA-based composite
TEF	Tetric EVO Flow
EHP	Enamel HRi Plus
AFM	atomic force microscope
RMS	root mean square
DC	direct current
rpm	rounds per minute
